# Supplementary material for: DMRforPairs: identifying Differentially Methylated Regions between unique samples using array based methylation profiles
Source: BMC Bioinformatics. 2014 May 15;15:141. doi: 10.1186/1471-2105-15-141 (PMC4046028; doi:10.1186/1471-2105-15-141)
Supplement: Additional file 2 — DMRforPairs output for the comparison of A431-MCF7 and NA17018-NA17105. Please start from the HTML files in each folder. Available via the BMC Bioinformatics website. [file 1471-2105-15-141-S2.zip › 1394847754114233_MOESM2_ESM/A431_MCF7/figures/11130.pdf]

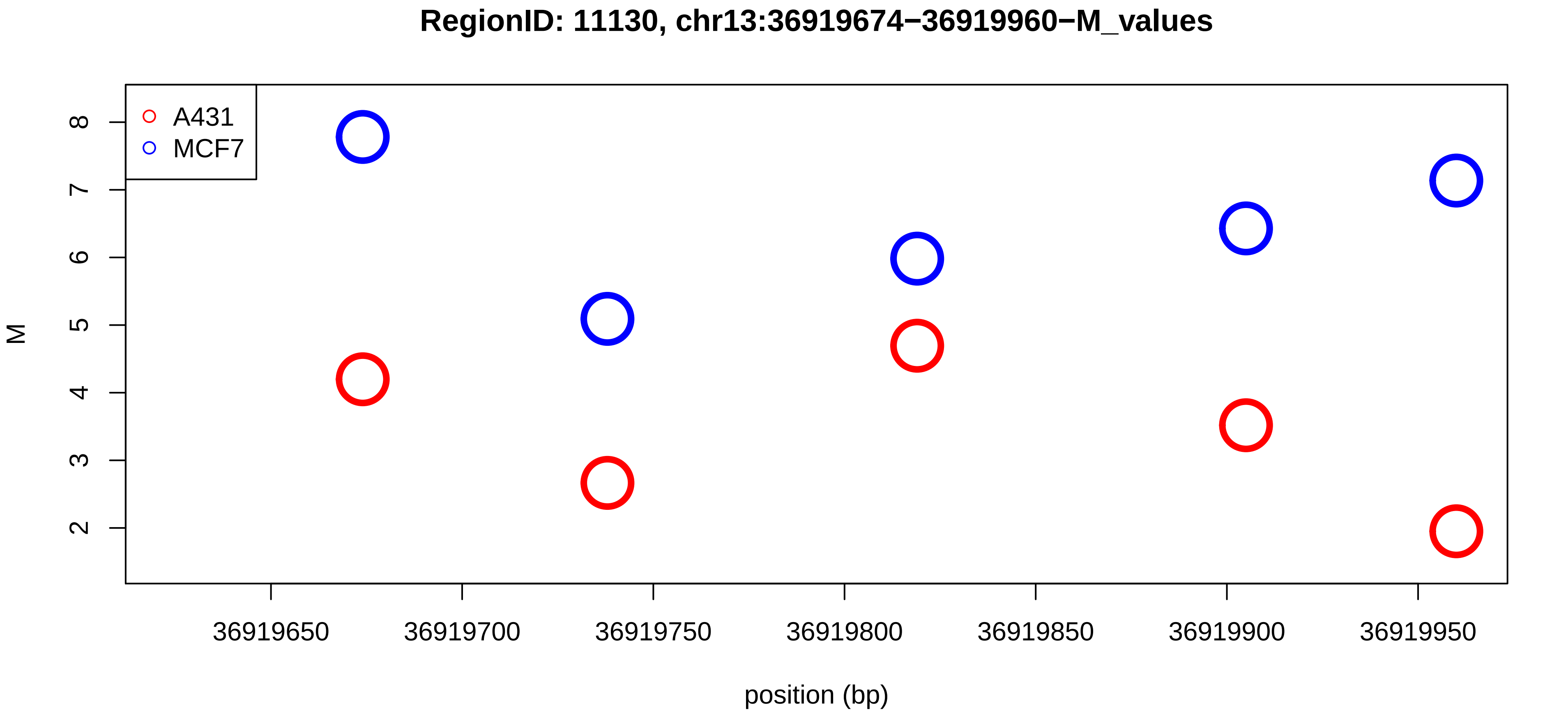
RegionID: 11130, chr13:36919674−36919960−M_values
A431
MCF7
M
2
3
4
5
6
7
8
36919650
36919700
36919750
36919800
36919850
36919900
36919950
position (bp)

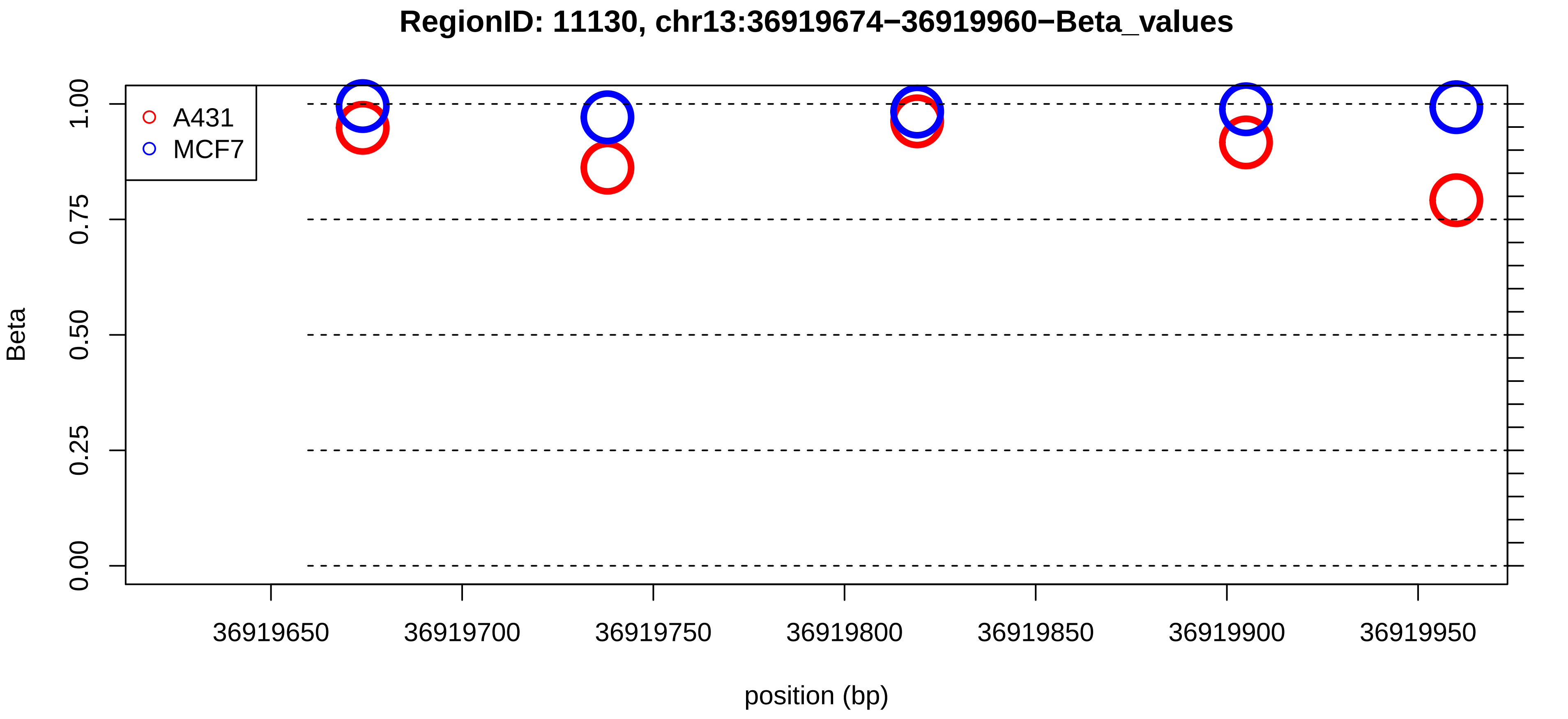
RegionID: 11130, chr13:36919674−36919960−Beta_values
A431
MCF7
Beta
0.00
0.25
0.50
0.75
1.00
36919650
36919700
36919750
36919800
36919850
36919900
36919950
position (bp)

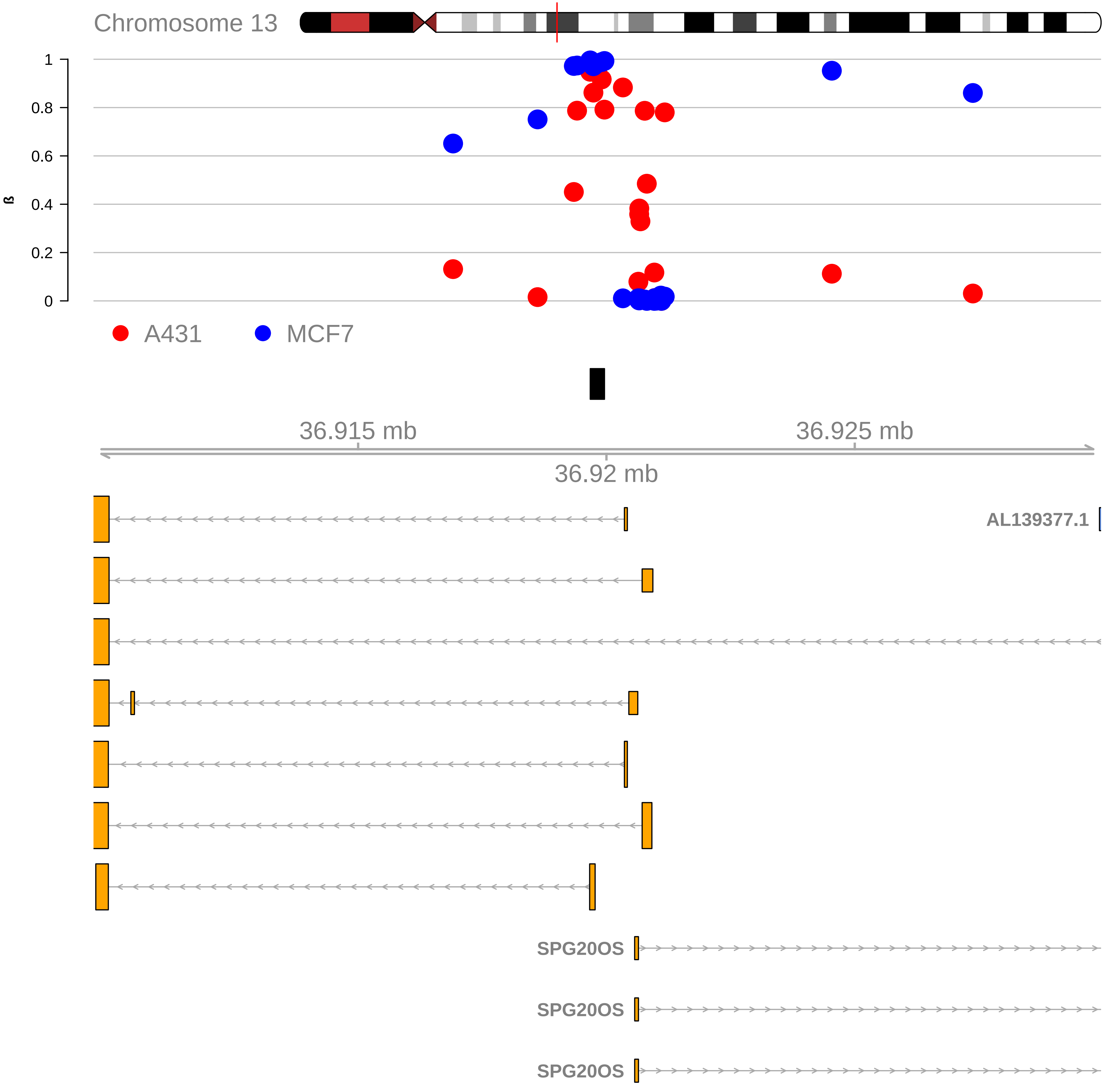
Chromosome 13
1
0.8
0.6
0.4
0.2
0
β
A431
MCF7
36.915 mb
36.925 mb
36.92 mb
AL139377.1
SPG20OS
SPG20OS
SPG20OS
